# Supplementary material for: Body height in young adult men and risk of dementia later in adult life
Source: eLife. 2020 Feb 11;9:e51168. doi: 10.7554/eLife.51168 (PMC7012597; doi:10.7554/eLife.51168)
Supplement: Supplementary file 1. [file elife-51168-supp1.docx]

| **Table S1** Distribution of covariates for body height and dementia diagnoses among the total population of men | | | |
| --- | --- | --- | --- |
|  | **Body height in cm** | **Dementia** | |
|  | **Mean (SD)** | **No N, (%)** | **Yes N, (%)** |
| **Intelligence level (deciles)** | | | |
| 1^st^ | 174.0 (6.6) | 64,381 (97.3) | 1,783 (2.7) |
| 2^nd^ | 175.1 (6.4) | 70,728 (98.0) | 1,476 (2.0) |
| 3^rd^ | 175.6 (6.4) | 61,380 (98.2) | 1,101 (1.8) |
| 4^th^ | 176.2 (6.4) | 70,687 (98.2) | 1,269 (1.8) |
| 5^th^ | 176.6 (6.4) | 57,912 (98.6) | 841 (1.4) |
| 6^th^ | 177.1 (6.4) | 80,365 (98.7) | 1,068 (1.3) |
| 7^th^ | 177.7 (6.4) | 59,156 (98.6) | 831 (1.4) |
| 8^th^ | 178.3 (6.4) | 70,967 (98.8) | 830 (1.2) |
| 9^th^ | 178.7 (6.4) | 54,072 (98.8) | 648 (1.2) |
| 10^th^ | 179.5 (6.3) | 60,682 (98.9) | 650 (1.1) |
| Missing | 177.0 (7.0) | 5,404 (98.2) | 102 (1.9) |
| **Educational level** | | | |
| Short | 175.0 (6.5) | 165,994 (98.1) | 3,282 (1.9) |
| Medium | 175.9 (6.3) | 213,337 (98.2) | 3,952 (1.8) |
| Long | 178.7 (6.4) | 270,352 (98.8) | 3,247 (1.2) |
| Missing | 176.7 (6.9) | 6,051 (98.1) | 118 (1.9) |
| **Birth cohorts** | | | |
| 1939-1944 | 175.2 (6.4) | 171,911 (97.0) | 5,338 (3.0) |
| 1945-1949 | 176.3 (6.5) | 151,927 (98.3) | 2,580 (1.7) |
| 1949-1954 | 177.4 (6.5) | 191,285 (99.0) | 1,903 (1.0) |
| 1955-1959 | 178.7 (6.5) | 140,611 (99.5) | 778 (0.6) |
| **Conscript board district** | | | |
| Copenhagen greater area incl. northern part of Zealand | 177.8 (6.7) | 202,091 (98.1) | 3,829 (1.9) |
| The remaining parts of Zealand and adjacent islands | 176.0 (6.6) | 94,369 (98.4) | 1,554 (1.6) |
| Funen and adjacent islands | 176.3 (6.5) | 54,728 (98.2) | 1,032 (1.8) |
| Southern and mid-eastern parts of Jutland | 176.9 (6.5) | 117,831 (98.8) | 1,472 (1.2) |
| North and West Jutland | 176.4 (6.4) | 115,775 (98.8) | 1,452 (1.2) |
| Bornholm | 175.3 (6.5) | 6,993 (98.4) | 114 (1.6) |
| The reunited southern part of Jutland | 176.4 (6.5) | 62,123 (98.2) | 1,112 (1.8) |
| Missing | 175.6 (6.2) | 1,824 (98.2) | 34 (1.8) |
