# Supplementary material for: Body height in young adult men and risk of dementia later in adult life
Source: eLife. 2020 Feb 11;9:e51168. doi: 10.7554/eLife.51168 (PMC7012597; doi:10.7554/eLife.51168)
Supplement: Supplementary file 2. [file elife-51168-supp2.docx]

| **Table S2** Mean body height (z-scores) in cm for each of the birth cohorts in the full study population, brothers and twins | | | |
| --- | --- | --- | --- |
|  | **Full study**  **population** | **Brothers** | **Twins** |
| **Birth cohorts** | **Body height (z-score) in cm** | | |
| **1939** | 175.0 (6.5) | 174.7 (6.8) | 173.3 (6.3) |
| **1940** | 174.5 (6.5) | 174.8 (6.34) | 173.0 (6.6) |
| **1941** | 174.7 (6.4) | 174.7 (6.6) | 172.9 (6.6) |
| **1942** | 175.2 (6.4) | 174.9 (6.3) | 174.4 (6.4) |
| **1943** | 175.6 (6.4) | 175.3 (6.5) | 173.8 (6.8) |
| **1944** | 175.8 (6.5) | 175.7 (6.6) | 174.1 (6.4) |
| **1945** | 175.9 (6.5) | 175.7 (6.3) | 174.8 (6.4) |
| **1946** | 176.3 (6.5) | 175.6 (6.5) | 175.1 (6.4) |
| **1947** | 176.6 (6.4) | 176.4 (6.4) | 175.9 (6.6) |
| **1948** | 176.6 (6.5) | 176.3 (6.5) | 175.7 (6.7) |
| **1949** | 176.7 (6.5) | 176.2 (6.5) | 175.5 (6.4) |
| **1950** | 176.9 (6.4) | 176.7 (6.4) | 175.8 (6.6) |
| **1951** | 177.4 (6.5) | 177.3 (6.5) | 176.4 (6.5) |
| **1952** | 177.8 (6.5) | 177.8 (6.5) | 176.5 (6.4) |
| **1953** | 177.8 (6.5) | 177.7 (6.5) | 177.1 (6.0) |
| **1954** | 177.6 (6.6) | 177.4 (6.6) | 176.5 (7.0) |
| **1955** | 178.3 (6.5) | 178.2 (6.5) | 177.9 (6.0) |
| **1956** | 178.6 (6.5) | 178.5 (6.6) | 178.1 (6.9) |
| **1957** | 178.7 (6.6) | 178.4 (6.6) | 178.0 (6.1) |
| **1958** | 178.8 (6.5) | 178.5 (6.5) | 177.6 (6.9) |
| **1959** | 179.1 (6.5) | 178.7 (6.5) | 179.3 (6.3) |
