# Supplementary material for: Body height in young adult men and risk of dementia later in adult life
Source: eLife. 2020 Feb 11;9:e51168. doi: 10.7554/eLife.51168 (PMC7012597; doi:10.7554/eLife.51168)
Supplement: Supplementary file 3. [file elife-51168-supp3.docx]

| **Table S3** Fully adjusted* hazard ratios (HRs) of the association between taller body height at entry to adulthood and dementia diagnosis among all men | |
| --- | --- |
| **Dementia cases**  **Number (%)** | **HR (95% CI) for onset of dementia per one z-score higher** |
| **<1995^¥^** | |
| 884 (0.1%) | 0.87 (0.82;0.93) |
| **≥1995^¥^** | |
| 9715 (1.6%) | 0.90 (0.89;0.91) |
| *Model 3: stratified by birth cohort and adjusted for conscript board district, educational level and intelligence test scores. Age included as underlying scale of the model.  ¥ These analyses follow men in different age range. The analyses of <1995 follow men from conscription up until a maximum age 36-56 years and the analyses of ≥1995 follow men from 36-56 years until a maximum age of 57-77 years | |
