# Supplementary material for: Body height in young adult men and risk of dementia later in adult life
Source: eLife. 2020 Feb 11;9:e51168. doi: 10.7554/eLife.51168 (PMC7012597; doi:10.7554/eLife.51168)
Supplement: Supplementary file 4. [file elife-51168-supp4.docx]

| **Table S4** Frequency (%) of individuals included from each birth cohort in the brother analyses | |
| --- | --- |
| **Birth cohort** | **Number (%)** |
| **1939** | 148 (0.2) |
| **1940** | 410 (0.6) |
| **1941** | 435 (0.6) |
| **1942** | 630 (0.9) |
| **1943** | 738 (1.1) |
| **1944** | 860 (1.2) |
| **1945** | 1,124 (1.6) |
| **1946** | 1,117 (1.6) |
| **1947** | 1,314 (1.9) |
| **1948** | 1,402 (2.0) |
| **1949** | 1,442 (2.0) |
| **1950** | 1,892 (2.7) |
| **1951** | 2,300 (3.3) |
| **1952** | 4,927 (7.0) |
| **1953** | 6,675 (9.5) |
| **1954** | 7,413 (10.5) |
| **1955** | 8,684 (12.3) |
| **1956** | 8,983 (12.7) |
| **1957** | 7,733 (11.0) |
| **1958** | 7,506 (10.6) |
| **1959** | 4,875 (6.9) |
