# Supplementary material for: Body height in young adult men and risk of dementia later in adult life
Source: eLife. 2020 Feb 11;9:e51168. doi: 10.7554/eLife.51168 (PMC7012597; doi:10.7554/eLife.51168)
Supplement: Transparent reporting form [file elife-51168-transrepform.docx]

***eLife’s* transparent reporting form**

We encourage authors to provide detailed information *within their submission* to facilitate the interpretation and replication of experiments. Authors can upload supporting documentation to indicate the use of appropriate reporting guidelines for health-related research (see [EQUATOR Network](http://www.equator-network.org/%20)), life science research (see the [BioSharing Information Resource](https://biosharing.org/)), or the [ARRIVE guidelines](http://www.plosbiology.org/article/info:doi/10.1371/journal.pbio.1000412) for reporting work involving animal research. Where applicable, authors should refer to any relevant reporting standards documents in this form.

If you have any questions, please consult our Journal Policies and/or contact us: [editorial@elifesciences.org](mailto:editorial@elifesciences.org).

**Sample-size estimation**

- You should state whether an appropriate sample size was computed when the study was being designed
- You should state the statistical method of sample size computation and any required assumptions
- If no explicit power analysis was used, you should describe how you decided what sample (replicate) size (number) to use

Please outline where this information can be found within the submission (e.g., sections or figure legends), or explain why this information doesn’t apply to your submission:

The Methods section includes information about the sample size and statistical methods. This study was based on cohort of 666,333 Danish men, given the large sample size, we did not conduct explicit power analysis.

**Replicates**

- You should report how often each experiment was performed
- You should include a definition of biological versus technical replication
- The data obtained should be provided and sufficient information should be provided to indicate the number of independent biological and/or technical replicates
- If you encountered any outliers, you should describe how these were handled
- Criteria for exclusion/inclusion of data should be clearly stated
- High-throughput sequence data should be uploaded before submission, with a private link for reviewers provided (these are available from both GEO and ArrayExpress)

Please outline where this information can be found within the submission (e.g., sections or figure legends), or explain why this information doesn’t apply to your submission:

Not applicable for this register-based cohort study.

**Statistical reporting**

- Statistical analysis methods should be described and justified
- Raw data should be presented in figures whenever informative to do so (typically when N per group is less than 10)
- For each experiment, you should identify the statistical tests used, exact values of N, definitions of center, methods of multiple test correction, and dispersion and precision measures (e.g., mean, median, SD, SEM, confidence intervals; and, for the major substantive results, a measure of effect size (e.g., Pearson's r, Cohen's d)
- Report exact p-values wherever possible alongside the summary statistics and 95% confidence intervals. These should be reported for all key questions and not only when the p-value is less than 0.05.

Please outline where this information can be found within the submission (e.g., sections or figure legends), or explain why this information doesn’t apply to your submission:

We have reported the statistical methods used in this study under the Methods section with the subheading Statistical analyses. All the results are provided under the Results section and in Table 1-3, Figure 1-2, Supplementary Table S1-4, Figure 1 – figure supplement 1 and Figure 2 – figure supplement 1.

(For large datasets, or papers with a very large number of statistical tests, you may upload a single table file with tests, Ns, etc., with reference to sections in the manuscript.)

**Group allocation**

- Indicate how samples were allocated into experimental groups (in the case of clinical studies, please specify allocation to treatment method); if randomization was used, please also state if restricted randomization was applied
- Indicate if masking was used during group allocation, data collection and/or data analysis

Please outline where this information can be found within the submission (e.g., sections or figure legends), or explain why this information doesn’t apply to your submission:

This study does not include experimental groups, so this information does not apply to this submission.

**Additional data files (“source data”)**

- We encourage you to upload relevant additional data files, such as numerical data that are represented as a graph in a figure, or as a summary table
- Where provided, these should be in the most useful format, and they can be uploaded as “Source data” files linked to a main figure or table
- Include model definition files including the full list of parameters used
- Include code used for data analysis (e.g., R, MatLab)
- Avoid stating that data files are “available upon request”

Please indicate the figures or tables for which source data files have been provided:

We have uploaded a file with the code used to program the statistical analyses performed in Stata. To access the anonymised dataset used in this study, researchers need to apply the Danish Conscript board steering committee and Statistics Denmark.  Only aggregated data, where no identification of persons is possible i.e. minimum five observations in each cell, can be removed from the server containing the data accessed through Statistics Denmark. Thus, we cannot provide an anonymised copy of the dataset as individuals may be identified based on the information in the data e.g. birthday, height, dementia status etc. Access to the data through Statistics Denmark is only granted for authorised research and analysis environments of a more permanent nature with a chief researcher and several researchers/analysts. Foreign researchers affiliated to a Danish authorised environment can also get access. Authorisation is granted by the Director General. Please find more information in the document ‘Access to micro data at Statistics Denmark_2014’ on <https://www.dst.dk/en/TilSalg/Forskningsservice>.
